# Responding to a Pandemic: The Role of EM-CCM on ICU Boarders in an Urban Emergency Department

**DOI:** 10.5811/westjem.2020.8.48671

**Published:** 2020-09-25

**Authors:** Jacqueline Pflaum-Carlson, Jayna Gardner-Gray, Namita Jayaprakash, Gina Hurst, Victor Coba, Harish Kinni, Emanuel Rivers

**Affiliations:** * Henry Ford Hospital, Department of Emergency Medicine, Detroit, Michigan; † Henry Ford Hospital, Division of Pulmonary and Critical Care Medicine, Detroit, Michigan; ‡ Henry Ford Hospital, Department of Surgery, Trauma and Surgical Critical Care, Detroit, Michigan

## INTRODUCTION

Severe acute respiratory syndrome coronavirus 2 (SARS-CoV-2), the novel coronavirus that was first detected in China, was declared a public health emergency of international concern on January 30, 2020. By March 11, 2020, the World Health Organization (WHO) characterized it as a global pandemic. The United States reported its first cases of coronavirus disease 2019 (COVID-19), the illness caused by SARS-CoV-2, on January 20, 2020. As of September 2, 2020, there have been over 6.26 million confirmed cases of COVID-19 in the United States with over 13,000 confirmed cases in the city of Detroit, Michigan.^1^ SARS-CoV-2 is a highly transmissible virus. The disease it causes, COVID-19, is a predominantly respiratory illness with varying symptom severity contributing to the potential for significant critical illness.

### The Setting

Henry Ford Hospital (HFH) is an urban, academic, quaternary referral center in Detroit. The hospital houses five distinct intensive care units (ICU) with 156 ICU beds; up to 68 of these beds can be used for medical intensive care unit (MICU) needs. In August 2016, the HFH emergency department (ED) established a Division of Emergency Medicine-Critical Care Medicine (EM-CCM) comprised of five specialty physicians with board certification in both EM and critical care medicine. The division has seen steady growth and faculty accrual each year. As the COVID-19 pandemic began in Detroit, the division of EM-CCM consisted of eight faculty who divide their clinical and non-clinical duties between the ED and the ICU. Additionally, these physicians form the early intervention team (EIT), working as critical care consultants in the ED and assisting with the delivery of focused management for critically ill patients. This includes post-resuscitative care, advanced ventilator management, adherence to ICU-bundled care, and selection and titration of vasoactive medications. As Michigan identified its first cases on March 10, 2020, the EM-CCM group at HFH found itself at the center of this overwhelming pandemic response.

### The Surge Response

#### Identifying the ED-ICU Needs

HFH ED includes a category 1 area: a hybrid ED-ICU zone that includes 16 beds and two resuscitation bays with the ability to care for and adapt to the management of incoming patients as well as ICU boarders. In early March, we learnt of the Italian experience with the surge of critical illness during COVID-19. It became clear that we would need to prepare to deliver early and prolonged critical care. Additionally, there was the consideration of isolation and protection of both patients and healthcare workers (HCW) from the transmission of SARS-CoV-2.^2^–^4^ This meant working with our ED and nursing leadership to adapt an area of category 1 into a COVID-19 ED-ICU where our ED nurses transitioned to essentially serve as both ED and ICU nurses.

#### Adapting our Setting

The number of cases presenting from the community rose rapidly in Detroit, requiring a shift in practice to presume that every patient with respiratory distress had COVID-19. This also meant a transition in ED workflow as we assumed many of our critical resuscitations would require aerosol-generating procedures (AGP) and performing AGPs in our positive-pressure airflow resuscitation bays would contribute to unnecessary HCW exposure. Thus, all resuscitations and AGPs were moved to negative pressure rooms and all resuscitation team members used personal protective equipment (PPE) for every resuscitation, including N95 masks. Moving resuscitation bays to negative pressure bays was disruptive; the transition required immense collaborative efforts between leadership, trauma services, nursing, ED technicians, and our ED pharmacists. Closed loop communication was essential as team members in PPE had to communicate needs to those outside the rooms. Fortunately, our hospital engineering facility team was able to convert the original resuscitation bays to negative air-flow spaces.

#### Expanding Early Intervention Team Coverage

Recognizing the potential for a surge of critically ill patients, the EIT identified the need for expanded coverage from five days a week (2 pm–10 pm), to seven days a week (2 pm–10 pm) and 24-hour on call availability. EIT performed “virtual rounds” and daily morning communications with the primary ED team. We also evaluated all ICU boarders for daily ICU rounding needs such as medication review, ventilator adjustments, and preventative care bundles. These virtual rounds allowed for enhanced communication with in-house ICU triage teams, prioritizing throughput based on severity of illness.

#### Guidelines and Procedures Evolving to Accommodate COVID-19

Developing guidelines during any pandemic response requires adaptability and rapid adjustment to changing standards of care. Responding to this particular pandemic, caused by a respiratory virus that had already resulted in a significant amount of critical illness and ventilator dependence worldwide, required prioritization in the protection of HCWs from accidental exposure and guidelines for decision-making when multiple, critically ill hypoxic patients arrive to the ED in tandem. As such, the EIT was critical in the development of ED-based guidelines that outlined and highlighted many of the necessary steps for protecting HCWs. Guidelines for patients undergoing AGPs stratified the risk of individual AGPs such as intubation, noninvasive positive-pressure ventilation (NIPPV), nebulizer treatments, and high-flow oxygen therapy, and recommended cohorting any patients suspected of having COVID-19 and undergoing an AGP into negative pressure rooms, followed by closed door rooms.

Ultimately, as the number of patients requiring AGPs grew, the ED heating, ventilation and air conditioning systems were re-engineered to support negative flow in larger areas allowing for safer cohorting of large groups of patients. Intubation guidelines focused on protection of HCWs by minimizing the use of bag valve mask (BVM) unless necessary, and the use of high efficiency particulate air (HEPA) filters if BVMs were needed. HEPA filters were also recommended for use on all NIPPV machines and ventilators. Cardiac arrest guidelines discussed details such as new positions for pharmacists outside resuscitations rooms to minimize exposure and use of PPE, the utilization of “runners” who acted as intermediaries between donned, code team members and the external ED team, communication recommendations using two-way radios, and avoiding patient disconnection from the vent during arrests to minimize aerosolization and HCW exposure.^5^ Lastly, EM-CCM physicians recognized that with rising numbers, community spread, and increasingly severe hypoxic presentations, resource limitations were inevitable.^6^ Thus, EIT advocated for management principles that would preserve access to invasive ventilation. This included the optimization of noninvasive oxygenation devices in appropriately ventilated rooms and, in some cases, participation in goals of care conversations with patients and their families while in the ED.

The novel nature of the virus meant that many of these guidelines relied heavily on experiences of healthcare systems in Europe and on the West Coast of the US, as well as prior experiences with severe acute respiratory syndrome and Middle East respiratory syndrome outbreaks; thus, the guidelines were updated and redistributed frequently. These ED guidelines, although originating for the ED from the Division of EM-CCM, were often translated hospital-wide via collaborative relationships of EM-CCM physicians with hospital committees and leadership.

### Reflections on the Pandemic Response

EM-CCM is a growing specialty with a unique perspective in the management of critically ill patients from the doors of the ED through the duration of their ICU stay. This perspective creates predictive insight into bottlenecks for admissions and discharges from ICUs, as well as a unique understanding of the adaptability and limitations of EDs during surges.

During the peak of the COVID-19 pandemic, HFH expanded its ICU capacities to eight ICUs primarily caring for COVID-19 patients. Despite this expansion, ED hold times were as long as 45 hours with daily averages between 6–12 hours. Prior to the pandemic, ICU boarding was not uncommon, but COVID-19 patients proved to be an additional challenge as they often required more advanced ventilator management strategies, higher doses of sedative medications, utilization of paralytics, and increased nursing monitoring and interaction. EIT physicians spent most of their time in full PPE moving from bedside to bedside, adjusting ventilator settings and reviewing medications to improve ventilator synchrony and oxygenation.

As the number of patients continued to rise, internal regulations for ICU requirements were adjusted to allow patients with high oxygen requirements, but not yet requiring high-flow nasal cannula or NIPPV, to be placed in general patient units (GPU). This conserved ICU beds, but meant more patients were being boarded in the lower acuity area of the ED while awaiting a GPU bed. As a result, EIT, already expanded in hours, expanded further into lower acuity areas of the ED to assess and monitor patients at risk for decompensation and ensure AGP and intubation guidelines were followed. The hybrid ED-CCM model at HFH proved ideal for this kind of adjustment, as the EIT physicians were able to expand beyond the physical ED-ICU space and assist in the wide spectrum of care of COVID-19 patients.

EM-CCM physicians leveraged their roles in surge committees and resuscitation councils to highlight the toll of COVID-ICU type care on the ED environment. Administrative leadership responded by halting all incoming transfers from outside hospitals and encouraging residents and fellows from other specialties to pick up ED shifts and to assist with screening and nasal swabs in a temporary tent facility. Critical care fellows were re-assigned from traditional electives to the EIT service in the ED to assist with critical care consultations and management. EIT physicians were involved in the development of a multidisciplinary proning team that was used both in the ED and the inpatient hospital setting to assist with the logistics of early proning. Finally, EIT used their relationship with anesthesia critical care physicians to set up an anesthesia procedure team to assist with ED intubations and procedures as we quickly realized that simultaneously managing the abundance of procedures while assessing newly arriving critically ill was an insurmountable task.

Outside of the ED, EIT’s presence in the ED and involvement in the care of nearly all patients who traveled through our ED into our ICUs, allowed the ICU to focus entirely on the patients within their units. This reduced the strain on ICU physicians for staffing, allowing more rest between COVID-19 ICU rotations. EIT physicians’ dual roles within the ED and the ICUs maintained clear lines of communication regarding ED and ICU needs during daily departmental town halls, allowing for early identification of resource scarcities within the hospital, and focused, bed-management discussions.

The HFH Division of EM-CCM continues to be included in the hospital ICU collaborative responses. The guidelines that were written initially for internal use were shared both systemwide and then with external ED leaders who reached out for assistance. During the COVID-19 pandemic response emergency physicians across the world stepped up to develop safe guidelines and protocols for the care of COVID-19 patients. At HFH, EM-CCM is a growing division that leveraged its position in both the EM and CCM worlds to help plan, prepare for, and support the surge of critically ill COVID-19 ICU boarders.
